# Overexpression of UHRF1 promoted the proliferation of vascular smooth cells via the regulation of Geminin protein levels

**DOI:** 10.1042/BSR20181341

**Published:** 2019-02-26

**Authors:** Xia Chen, You-li Zhou, Shi-yu Liang, Yan-chuan Shi, Shu Lin, Mao-qin Shu

**Affiliations:** 1 Department of Cardiology, Southwest Hospital, Third Military Medical University (Army Medical University), China; 2 Neuroscience Division, Garvan Institute of Medical Research, 384 Victoria Street, Darlinghurst, Sydney, NSW 2010, Australia; 3 School of Health Science, IIIawarra Health and Medical Research Institute, University of Wollongong, NSW 2522, Australia

**Keywords:** Geminin, PI3K-Akt signaling pathway, UHRF1, VSMCs

## Abstract

Geminin is an inhibitor of DNA replication licensing and cell cycle. Our previous study demonstrates that Geminin plays an important role in regulating phenotypic diversity and growth of vascular smooth cells (VSMCs). Ubiquitin-like with PHD and RING Finger domains 1 (UHRF1) is an epigenetic coordinator, whose RING domain confers intrinsic E3 ligase activity, mediating the ubiquitination of several proteins and the protein–protein interaction. Aberrant expression of UHRF1 was related to aggressiveness of multiple human malignancies, where knockdown of UHRF1 led to decreased proliferation of cancer cells. However, it is unclear whether proper UHRF1 function is involved in aberrant proliferation and phenotypic switching of VSMCs via altering Geminin protein levels. In present study, in UHRF1-overexpressing A10 cells, 3H-thymidine and 5-ethynyl-20-deoxyuridine (EdU) and CCK8 were used to examine the proliferation of VSMCs. RT-PCR and Western blot analyses were performed to investigate whether UHRF1-mediated effects were achieved by altering Geminin expression in VSMCs. RNA-seq analysis was performed to dissect related mechanisms or signaling pathways of these effects. The results of *in vitro* experiments suggested that UHRF1 prompted proliferation and cell cycle of VSMCs via the down-regulation of Geminin protein levels with no change in Geminin mRNA expression. Besides, PI3K-Akt signaling pathway was increased upon UHRF1 up-regulation. Our study demonstrated that overexpressing UHRF1 was involved in VSMCs proliferation through reducing inhibitory Geminin protein levels to promote cell cycle as well as activating PI3K-Akt signaling. This may provide key knowledge for the development of better strategies to prevent diseases related to VSMCs abnormal proliferation.

## Background

In response to vascular injury, the vascular smooth muscle cells (VSMCs) undergo the phenotypic changes from the contractile type (differentiation, rarely proliferate) to the synthetic type (dedifferentiation), which is also accompanied with altered expression of proliferation markers in VSMCs [1–3]. In atherosclerotic lesions, chronic inflammation induces aberrant phenotypic switching of VSMCs that leads to increased de-differentiation and proliferation that contribute to the progression of atherosclerosis [4,5]. Our previous study found that Geminin, an inhibitor of DNA replication licensing and cell cycle, played an important role in phenotypic diversity and growth of VSMCs [6,7]. We observed that overexpression of Geminin stimulated VSMCs to switch from the de-differentiation to the differentiation type, while down-regulation of Geminin promoted VSMCs proliferation and G1/S cell-cycle progression. Intriguingly, in the contractile type and the synthetic type with different proliferative potentials, there was no significant difference with regards to Geminin mRNA levels [6,7], which suggested that it could be the regulation of Geminin protein expression that mediates the effects on the phenotypic switching and proliferation of VSMCs.

Ubiquitin-like with PHD and RING Finger domains 1 (UHRF1), an epigenetic coordinator, functionally regulates DNA methylation, DNA repair, chromatin modifications, cellular proliferation and cell cycle. UHRF1 mainly acts through five recognizable domains including N-terminal Ub-like, tudor, PHD, SRA and RING domains to participate in these processes [8–10]. It is worth noting that the RING domain of UHRF1 confers intrinsic E3 ligase activity, which mediates the ubiquitination of several proteins and protein–protein interaction [11–13]. Besides, the ligase activity of UHRF1 also plays a critical role in proliferation of cells under different environmental stresses [12]. Previous studies showed that UHRF1 was highly expressed in proliferating cells and inevitably required for G1/S phase transition [14,15]. In addition, as Geminin levels change over the cell cycle [16], mRNA and protein levels of UHRF1 fluctuate with the cell cycle, and depletion of UHRF1 inhibits S phase entry [17–19]. Collectively, we proposed that proper UHRF1 function may be required in aberrant proliferation and phenotypic switching of VSMCs via altering Geminin protein levels. In the present study, we tested the hypothesis by overexpressing UHRF1 in contractile type VSMCs and investigated how UHRF1 regulated the VSMCs proliferation and cell cycle.

## Materials and methods

### VSMCs culture

The thoracic aortic smooth muscle cell line of rats, A10 cells (ATCC, U.S.A.) were cultured in Dulbecco’s modified essential medium (DMEM) (Gibco, U.S.A.) supplemented with 10% fetal bovine serum (FBS) (Gibco, U.S.A.) and a 1% penicillin/streptomycin in a humidified atmosphere (5% CO_2_, 95% air). The contractile type and synthetic type of VSMCs were induced by 0.5% and 10% serum, respectively [1,7,20]. The morphology and protein markers were examined as previously described [6]. Cells from passages 3 through 6 were used in all experiments.

### Construction of UHRF1 recombinant lentiviral vectors

The UHRF1 gene overexpression lentivirus (LVs) was constructed and purchased from Obio Technology (Shanghai) Co. Ltd. (Shanghai, China). The full length of human UHRF1 gene (NCBI reference sequence ID, NM_001008882), which was marked by Puro, was encoded into H156 vector. The empty vector (LV-empty-*Puro*) and LV with *UHRF1* (LV-*UHRF1-Puro*) were co-transfected into 293T cells in serum-free medium using Lipofectamine 2000 (Invitrogen Inc., U.S.A.). The medium was changed to complete medium (DMEM supplemented with 10% FBS) after 8 h of incubation. High-titer recombinant lentiviral vectors carrying UHRF1 were harvested after 48 h of transfection.

### Transfection

A10 cells in log phase were divided into three groups. One group of cells was planted at a density of 1 × 10^4^ cells/well in 96-well plates and transfected with UHRF1-Puro vectors (*UHRF1-high group*, MOI was 100-150) in serum-free medium. Polybrene (5 μg/ml) was added to improve transfection efficiency as an enhancing reagent. After 8 h, the medium was changed to complete medium. The second group of cells was transfected with empty-Puro lentiviral vectors (empty-Puro group, negative control) in the same manner as UHRF1-Puro group. The third group of cells without intervention served as the blank control group. After 3 days of transfection, cells selected with puromycin (1–10 μg/ml) were harvested for further experiments.

### RNA isolation and real-time PCR

RNAiso Plus (Takara, Japan) was used to extract total RNA from the cultured cells according to the manufacturer’s instructions. Total RNA was reversely transcribed into cDNA using PrimeScriptTM RT reagent Kit (Perfect Real Time, Takara, Japan). Real-time quantitative polymerase chain reaction (qPCR) analysis for UHRF1 and Geminin was performed using One-Step SYBR® PrimeScriptTM RT-PCR Kit (Perfect Real Time) and the ΔΔ*C*_t_ method with housekeeping gene GAPDH as the endogenous control (the primer sequences for the genes were presented in Table 1). Melting curve analysis was performed to confirm the exclusive amplification of the expected PCR product.

**Table 1:** Primer sequences for gene expression analyzed by qRT-PCR

Target Gene	Forward (5’-3’)	Reverse (5’-3’)
GAPDH	TACCCACGGCAAGTTCAA	GCCAGTAGACTCCACGACAT
UHRF1	ATGACTCTACCCACGGCAAG	CTGGAAGATGGTGATGGGTT
Geminin	GAGCCCAAGAGAACGTGAAGAGTAG	CCTCCGTTGTTCTGCCACTTCTTTC

### Western blotting

Total proteins were extracted from cultured cells and the protein concentration was measured by BCA kit (Beyotime). Equal amounts of protein lysates were separated on a 10% SDS-PAGE, transferred to PVDF membrane and then immunoblotted with antibodies against Geminin (1:1000 dilution; Cell Signaling Technology, #5165, Danvers, U.S.A.), UHRF1 (1:1000 dilution; Abcam, ab213223, Cambridge, U.K.) and β-actin (1:5000 dilution; Absci, AS014, Nanjing, China), followed by incubating with HRP goat anti-rabbit IgG (ABclonal, Wuhan China). As for UHRF1 and Geminin, the dilution of the HRP goat anti-rabbit IgG antibody was 1:2000; as for β-actin, the dilution was 1:10,000. The primary antibody UHRF1, Geminin and β-actin were incubated at 4°C overnight. The second-antibody HRP goat anti-rabbit IgG was incubated at room temperature for 2 h. The results were visualized using Image Lab Imaging System software.

### CCK8 assay

VSMCs were plated at a density of 8000 cells/well in 96-well plates in a 37°C, 5% CO_2_. When grown to 70–80% confluence, cells were synchronized in serum-free DMEM for 24 h and then incubated with 0.5% serum. After 24 h of 0.5% serum stimulation, 20 μl of CCK8 cell count kit (Boster, Wuhan China) was added to cells for 4 h, followed by light absorbance measurement at a wavelength of 450 nm.

### Measurement of EdU incorporation

VSMCs were plated in 96-well plates and synchronized as described above in CCK8 assay. After 24 h of 0.5% serum stimulation, A10 cell proliferation was determined by the 5-ethynyl-20-deoxyuridine (EdU) Cell Proliferation Assay Kit (Ribobio, Guangzhou, China), which was performed according to the manufacturer’s protocol. The cell nuclei were stained with DAPI (1:100; Beyotime, Shanghai, China), and the incorporated EdU in A10 cells was detected by fluorescence microscopy.

### Cell cycle analysis

We performed the Cell cycle analysis according to the manufacturer’s protocol of the cell cycle assay kit (Bestbio, Shanghai, BB-4104). VSMCs were seeded in the six-well plates with 500,000 cells/well and cultured in 0.5% serum for 24 h. After starvation using serum-free media for 24 h, the cells were incubated with 0.5% serum for 24 h. Then VSMCs were fixed in 70% ethanol and incubated overnight at −4°C. The cell cycle was analyzed by propidium iodide (PI) and determined by a flow cytometer (Beckman Coulter).

### RNA-seq analysis

An RNA-Seq library was constructed for UHRF1 overexpression group (*UHRF1-Puro* A10 cells), negative control group (empty-*Puro* A10 cells) and blank control group (A10 cells), which was sequenced following the Illumina HiSeq2000 protocol to generate 90-bp paired-end reads. Genes with at least 10 mapped reads were considered as reliably detected genes. The quality of the RNA-Seq reads from all samples were assessed using Agilent Bioanalyzer 2100. Genome mapping was performed on the pre-processed reads using the spliced mapping algorithm of Hisat2 (v 2.0.4). The number of mapped reads was counted using Stringtie (version 1.3.0).

### Data analysis

Data were analyzed using GraphPad Prism 6 with Student’s *t*-test. *P*<0.05 was considered statistically significant.

## Results

### UHRF1 is highly expressed in synthetic type of VSMCs

**Figure 1:**
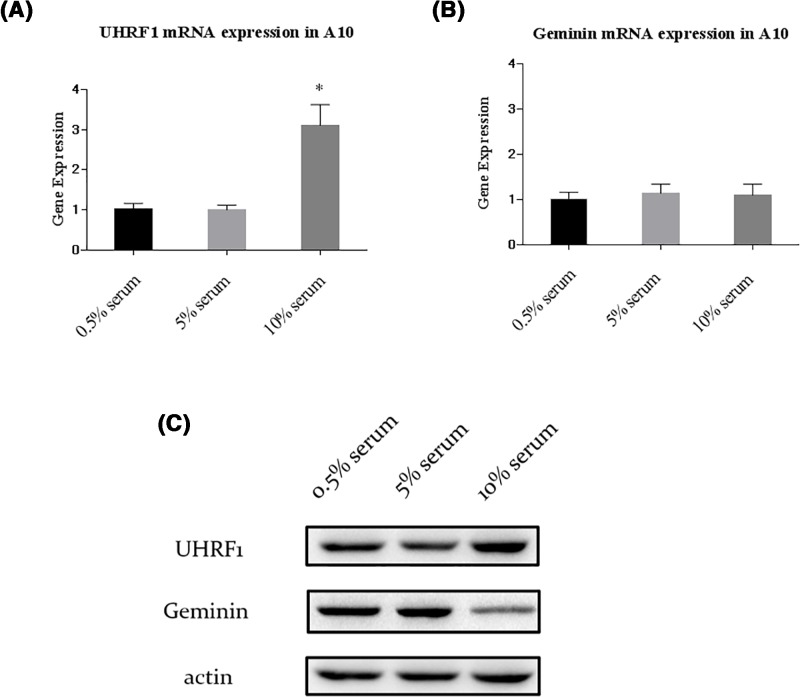
UHRF1 and Geminin expression in VSMCs (**A**) mRNA expression levels of UHRF1 in 0.5%, 5% and 10% serum incubated VSMCs, *P<0.05 versus 0.5% serum group. (**B**) mRNA expression levels of Geminin in 0.5%, 5% and 10% serum incubated VSMCs, there was no significant difference of Geminin mRNA expression among these three groups. (**C**) Protein levels of UHRF1 and Geminin in 0.5%, 5% and 10% serum incubated VSMCs. All values are means from three independent experiments.

It has been demonstrated that the contractile type and synthetic type of VSMCs were induced by 0.5% and 10% serum respectively [7]. To determine whether UHRF1 expression was changed in different phenotypes and proliferation of VSMCs, the A10 cells were incubated in media containing 0.5% serum, 5% serum and 10% serum respectively for 24 h (we also tested the protein and mRNA expression levels of Geminin and UHRF1 in A10 cells incubated for 12 and 36 h. The more details of the results were presented in Supplementary Figures S1 and S2). As shown in Figure 1A,B, UHRF1 mRNA expression was highly expressed in the synthetic type, while there was no difference in Geminin mRNA levels among three groups. Results from Western blot indicated that UHRF1 protein levels were moderately higher than those in synthetic type, which was consistent with the data of qPCR analysis (Figure 1C and Supplementary Figure 3S). Interestingly, compared with 0.5% and 5% serum groups, protein expression of Geminin in 10% serum was markedly decreased in the synthetic type. Given the effects of Geminin on VSMCs, these results imply that UHRF1 may have a role in the regulation of VSMCs phenotype and proliferation.

### Overexpression of UHRF1 promoted the proliferation of VSMCs contractile type

**Figure 2:**
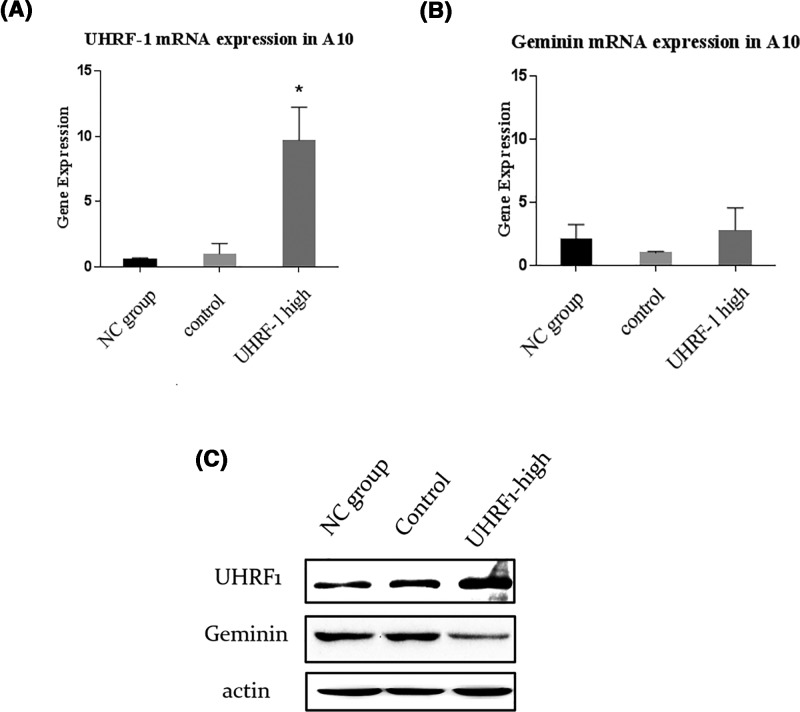
UHRF1 and Geminin expression in VSMCs cultured in 0.5% serum (**A**) mRNA expression levels of UHRF1 in NC group, control and UHRF1-high group, *P<0.05 versus 0.5% serum group. (**B**) mRNA expression levels of Geminin in NC group, control and UHRF1-high group, there was no significant difference of Geminin mRNA expression among these three groups. (**C**) Protein levels of UHRF1 and Geminin in NC group, control and UHRF1-high group. All values are means from three independent experiments.

**Figure 3:**
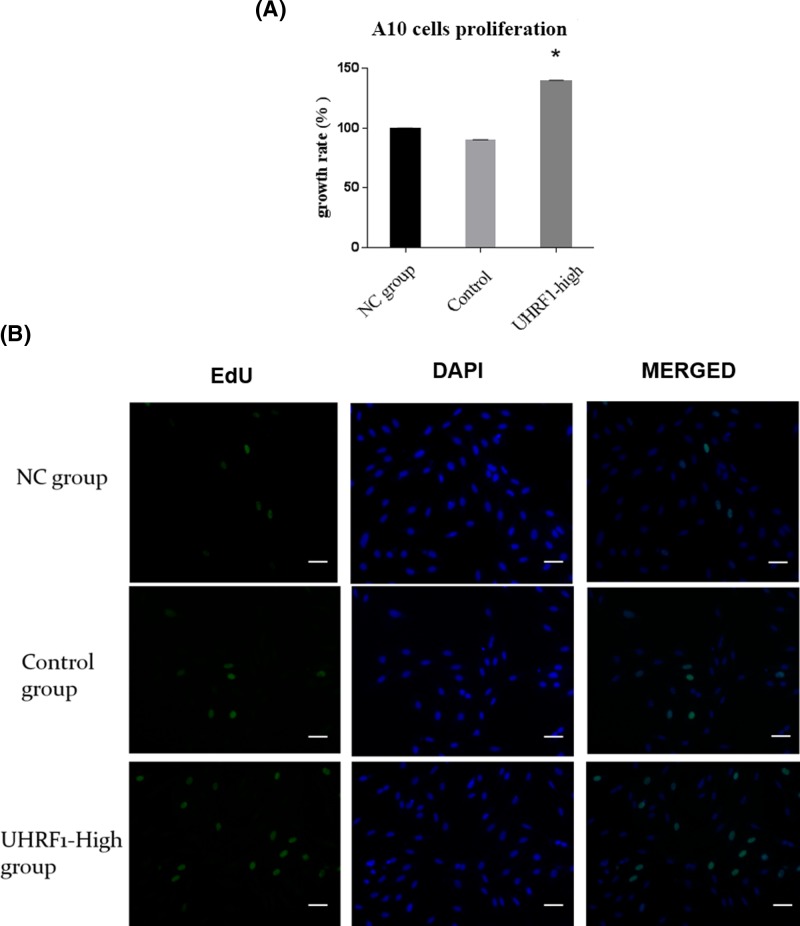
Effects of overexpression UHRF1 on the proliferation of VSMCs contractile type (**A**) NC group, control and UHRF1-high group were stimulated with 0.5% serum for 24 h. An index of cell proliferation was analyzed by CCK8 assay. The growth rate is normalized to the NC group. *P<0.05 versus vehicle group. The values of each group are means from 12 samples. (**B**) EdU staining assay analysis. The cells with green fluorescence indicate cells undergoing proliferation, and the cells with blue fluorescence represent all the cells. All groups were incubated with 0.5% serum for 24 h. Scale bars are 20 μm in all images.

Numerous studies have proven that UHRF1 expression is up-regulated in a variety of cancer cells, which drives the malignant growth [21–23]. In our study, in order to investigate whether overexpression of UHRF1 facilitated VSMCs proliferation, lentivirus was used to establish the *UHRF1-High* A10 models that up-regulated UHRF1 at both mRNA and protein levels. The total RNA and protein were extracted from NC (Normal control group), empty-*Puro* and *UHRF1-Puro* A10 cells cultured in 0.5% serum, and results of qPCR and Western blot analysis confirmed a significant increase in UHRF1 gene expression at both mRNA and protein levels in the *UHRF1-Puro* transfected cells (Figure 2A,C). As a result, we observed increased growth rate as well as EdU staining in the same treatment groups (Figure 3B and Supplementary Figure 4S) (*P<0.05 vs. NC group, Figure 3A), indicating that overexpression of UHRF1 promoted DNA synthesis, thereby increasing the growth of A10 cells. Thus, we concluded that UHRF1 may prompt proliferation of VSMCs of contractile type.

### UHRF1 was involved in the regulation of Geminin protein levels in VSMCs contractile type

Considering that Geminin has an important role in VSMCs proliferation and Geminin was highly expressed in VSMCs contractile type, we explored whether the proliferative effect of UHRF1 in VSMCs of contractile type is related to Geminin expression. The total RNA and protein were extracted from the cells cultured in 0.5% serum. As shown in Figure 2B, there was no significant difference in the mRNA levels of Geminin among these three groups. However, compared with NC group and empty-*Puro* group, Geminin protein levels sharply decreased in *UHRF1-Puro* transfected cells (Figure 2C), which indicates that up-regulation of UHRF1 may inhibit Geminin protein expression but not mRNA expression to promote growth of VSMCs of contractile type.

### Overexpression of UHRF1 promoted the cell cycle progression of VSMCs contractile type

**Figure 4:**
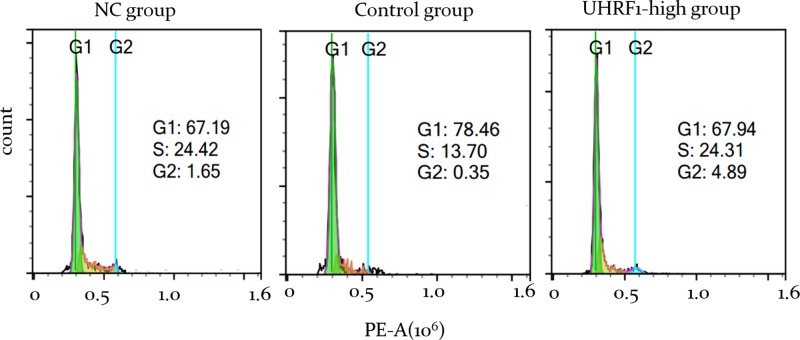
Overexpression of UHRF1 promotes cell cycle progression in VSMCs contractile type All groups were incubated in 0.5% serum and harvested at 24 h. The cells were labeled with propidium iodide (PI). Cell samples were analyzed by using a 488-nm excitation wavelength and a 610-nm emission wavelength. Triplicate samples of each group were analyzed at the same time.

Given that Geminin is an inhibitor of DNA replication licensing and cell cycle, we evaluated whether overexpression UHRF1-induced changes of Geminin protein expression play a role in cell cycle progression of VSMCs contractile type. We performed the flow cytometry on A10 cells (*UHRF1-Puro* group, empty-*Puro* group and NC group) incubated in 0.5% serum for 24 h. In *UHRF1-Puro* group, more cells were in G2 phases compared with NC group at the same time point (1.65% vs. 4.89% at 24 h) (Figure 4). It has been reported that Geminin prevents DNA replication at S phase and induces cell cycle arrest in S phase [24,25]. Thus, the enhancing effect of UHRF1 on the proliferation of VSMCs may act through the down-regulation of Geminin, thereby promoting cell cycle progression.

### UHRF1-stimulated growth of VSMCs contractile type is related to PI3K-Akt signaling pathway

**Figure 5:**
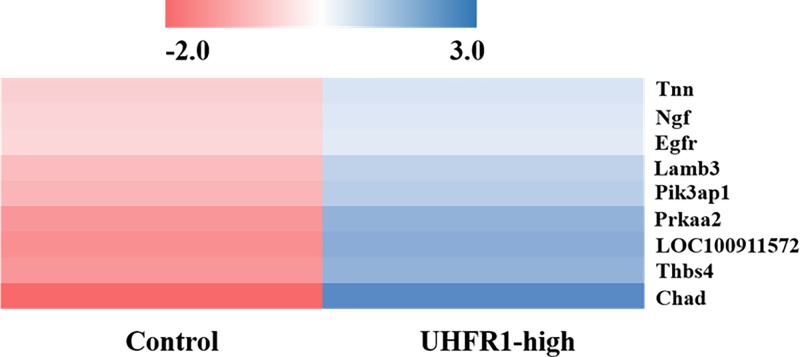
Genes activated in empty-*Puro* and *UHRF1-Puro* A10 cells Hierarchical clustering of representative PI3K-Akt signaling genes that were up-regulated in UHRF1-high A10 cells.

In order to identify the possible involvement of other mechanisms or signaling pathways through which UHRF1 regulates the growth of VSMCs contractile type, we conducted RNA-seq analysis to thoroughly profile the global gene expression of VSMCs in *UHRF1-Puro* group and NC group. We identified 163 genes with at least 2-fold changes upon UHRF1 up-regulation. The expression levels of Lamb3, Pik3ap1, Prkaa2 and Thbs4 genes that participate in PI3K-Akt signaling pathway were significantly increased upon UHRF1 overexpression (Figure 5 and Supplementary Table S1), suggesting that this pathway that is critical in regulating the cell cycle and directly related to cellular quiescence, proliferation and longevity was activated by UHRF1 overexpression.

## Discussion

Previous studies have demonstrated that UHRF1 was highly expressed in proliferating cells and inevitably required for G1/S phase transition [17]. Aberrant expression of UHRF1 was related to aggressiveness of multiple human malignancies, whereas knockdown or silencing of UHRF1 in cancer cells led to decreased proliferation and increased apoptosis [15]. In the present study, UHRF1 expression was measured in VSMCs contractile type and synthetic type, respectively. We found that UHRF1 levels were higher in VSMCs synthetic type with proliferation potential, which suggests that UHRF1 is crucial in VSMCs proliferation and phenotypic transformation. By using UHRF1-overexpression A10 cells, we first dissected the function and underlying mechanism of UHRF1 in VSMCs proliferation. *In vitro* experiments suggested that UHRF1 prompted proliferation and cell cycle of VSMCs contractile type via down-regulation of Geminin protein levels without changing Geminin mRNA expression.

Geminin and UHRF1 levels both fluctuated during the cell cycle. In normal rat VSMCs, we observed that protein levels, not mRNA levels of Geminin were higher in contractile type than synthetic type. Similarly, when overexpressing UHRF1 in VSMCs contractile type, it was also not mRNA but Geminin protein levels sharply decreased in these cells. Collectively, UHRF1 modulates Geminin protein expression but not mRNA expression to promote growth of VSMCs contractile type. Protein degradation is mediated by proteases, among which a set of enzymes termed E1, E2 and E3 mediate the conjugation of ubiquitin and consequently confirm short half-lives on proteins to be ubiquitylated [26]. UHRF1 contains a RING finger domain at its C-terminus, with intrinsic E3 ligase activity toward histones and non-histone proteins. For instance, it has been reported that UHRF1 interacts with TIP60 (Tat-interacting protein of 60 kDa, a member of lysine acyltransferases) and induces degradation-independent ubiquitination of TIP60 [27]. Moreover, previous studies demonstrate that UHRF1 with RING-dependent E3 ubiquitin ligase activity could play a vital role in assembly of a protein complex required for cellular proliferation [12]. Here, we reported, for the first time, that UHRF1 regulated the protein expression of Geminin and promoted proliferation in VSMCs, and the effects may be related to E3 ubiquitin ligase activity of UHRF1. Mechanistically, the PI3K-Akt pathway is a key regulator of cell survival, and hyperactivation of the PI3K-Akt pathway promotes proliferation and survival of cancer cells [28,29]. In addition, embryonic stem cells require active PI3K-Akt signaling to maintain their undifferentiated properties [30]. In the present study, RNA-seq analysis revealed that PI3K-Akt signaling pathways was activated in *UHRF1-Puro* group. These data suggested that PI3K-Akt signaling could participate in abnormal growth of VSMCs. However, further investigations are required to clarify whether UHRF1 directly activates PI3K-Akt signaling, or activates this pathway through its ubiquitin ligase activity, thereby promoting VSMCs proliferation.

## Conclusion

In general, UHRF1 can be a potential cancer drug target, whose overexpression correlates with aggressiveness of multiple human malignancies. Knockdown or silencing of UHRF1 in cancer cells leads to reduced proliferation and increased apoptosis. Notably, our study demonstrate that overexpressing UHRF1 was also involved in VSMCs proliferation through reducing inhibitory Geminin protein levels to promote cell cycle as well as activating PI3K-Akt signaling. This may provide a key knowledge for the development of treatments for atherosclerosis, which would help develop better strategies to prevent diseases related to VSMCs abnormal proliferation.

## Supporting information

**Table S2A:** statistical data of Figure1A

## Abbreviations

TIP60: Tat-interacting protein
UHRF1: ubiquitin-like with PHD and RING Finger domains 1
VSMC: vascular smooth muscle cell
