# Supplementary material for: Overexpression of UHRF1 promoted the proliferation of vascular smooth cells via the regulation of Geminin protein levels
Source: Biosci Rep. 2019 Feb 26;39(2):BSR20181341. doi: 10.1042/BSR20181341 (PMC6390124; doi:10.1042/BSR20181341)
Supplement: Supplementary file 1 [file bsr-39-bsr20181341_Supp1.pdf]

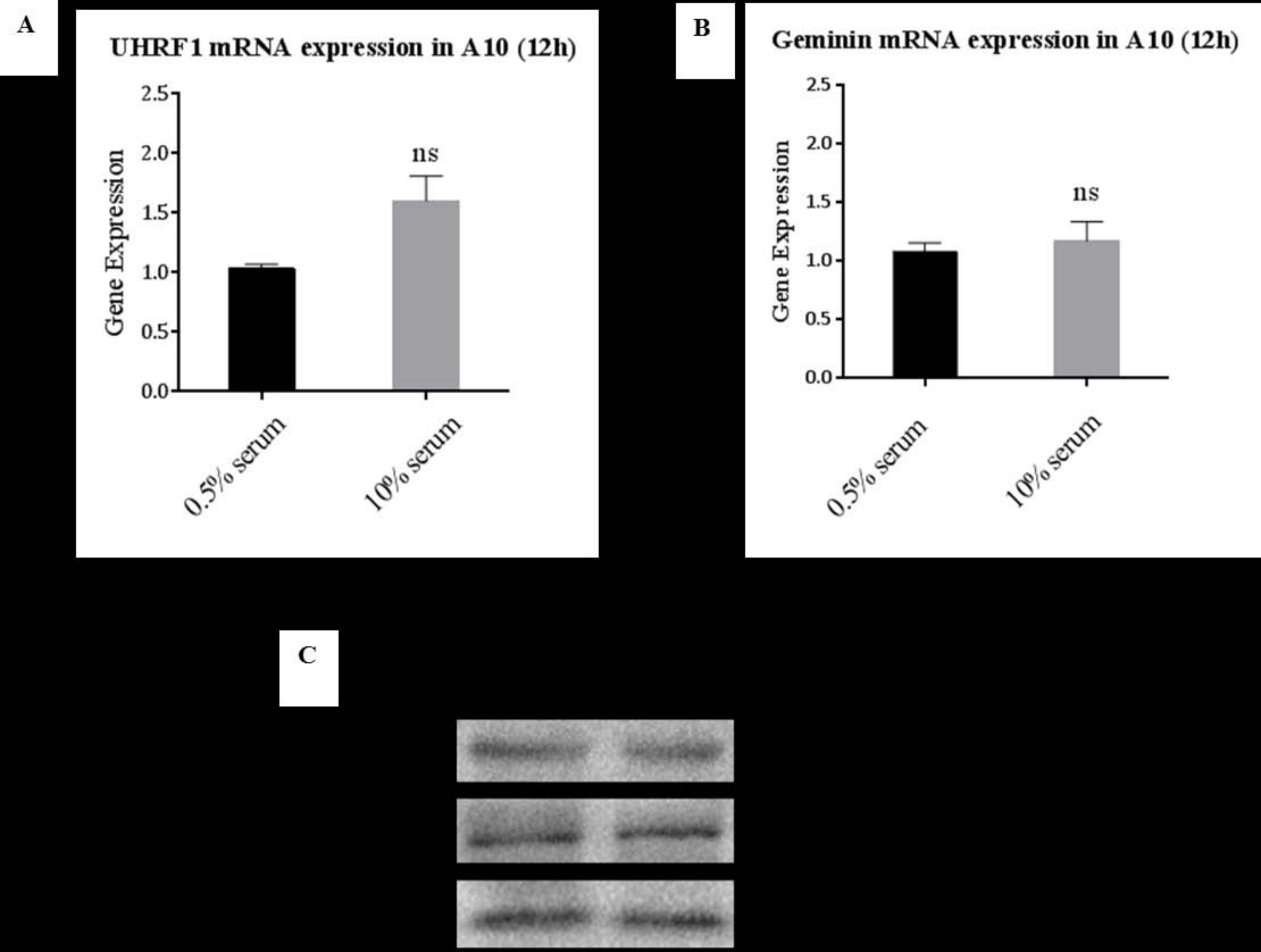
A
UHRF1 mRNA expression in A10 (12h)
Gene Expression
2.5
2.0
1.5
1.0
0.5
0.0
ns
0.5% serum
10% serum
B
Geminin mRNA expression in A10 (12h)
Gene Expression
2.5
2.0
1.5
1.0
0.5
0.0
ns
0.5% serum
10% serum
C

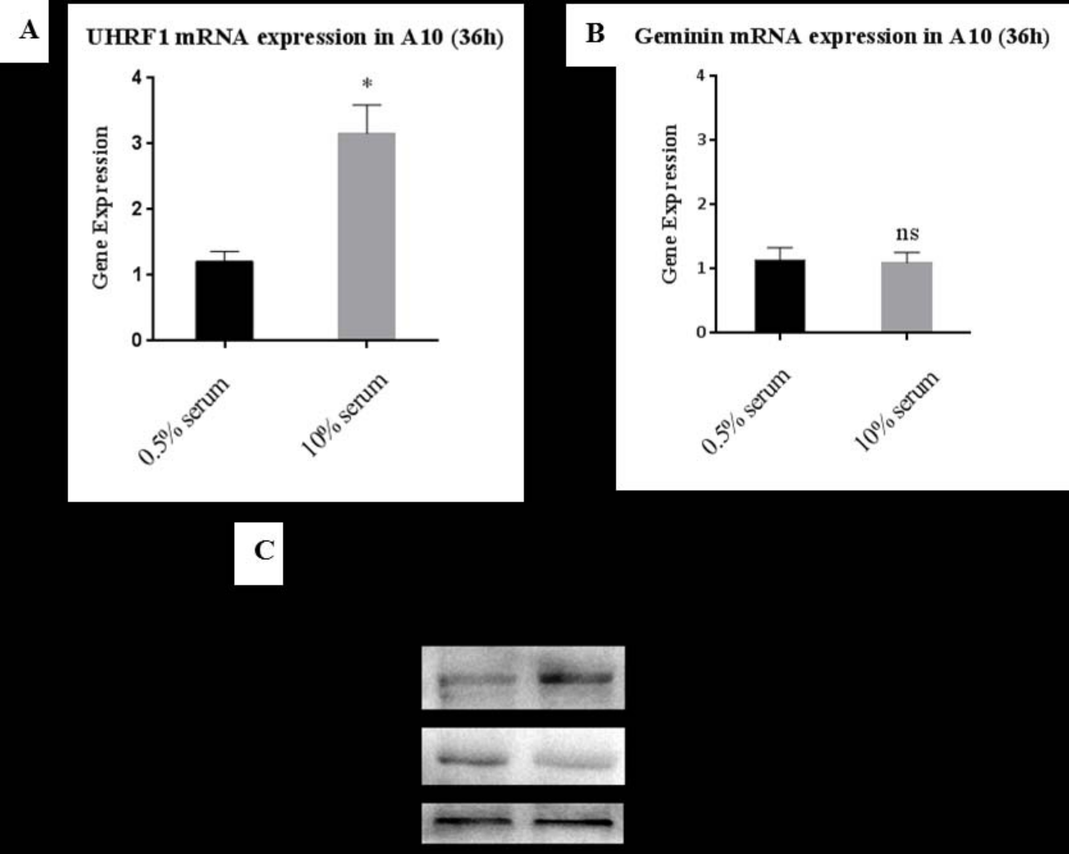
A
UHRF1 mRNA expression in A10 (36h)
Gene Expression
0
1
2
3
4
*
0.5% serum
10% serum
B
Geminin mRNA expression in A10 (36h)
Gene Expression
0
1
2
3
4
ns
0.5% serum
10% serum
C

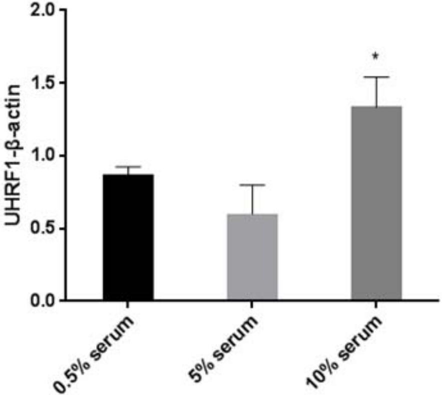
2.0
1.5
1.0
0.5
0.0
UHRF1-β-actin
*
0.5% serum
5% serum
10% serum

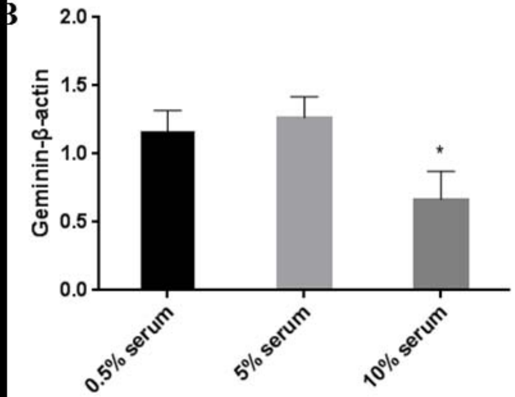
B
2.0
1.5
1.0
0.5
0.0
Geminin-β-actin
*
0.5% serum
5% serum
10% serum

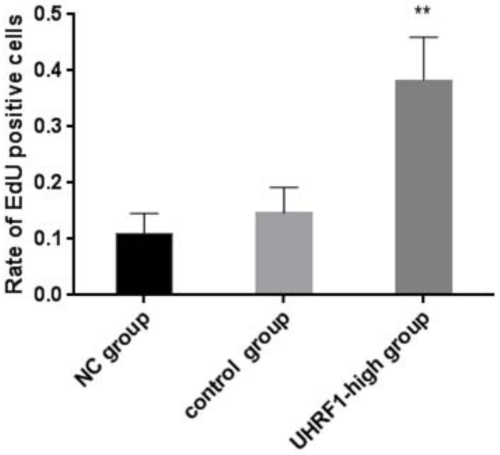

Rate of EdU positive cells
0.5
0.4
0.3
0.2
0.1
0.0
**
NC group
control group
UHRF1-high group

Table S2A statistical data of Figure1A

NC group			Control group			UHRF1-high group		
**Means**	**SD**	**N**	**Means**	**SD**	**N**	**Means**	**SD**	**N**
1.02269	0.13930	3	1.00136	0.12117	3	3.10779	0.51793	3

Table S2B statistical data of Figure1B

NC group			Control group			UHRF1-high group		
**Means**	**SD**	**N**	**Means**	**SD**	**N**	**Means**	**SD**	**N**
1.02269	0.13930	3	1.13859	0.20196	3	1.09663	0.24588	3

Table S2C statistical data of Figure2A

NC group			Control group			UHRF1-high group		
**Means**	**SD**	**N**	**Means**	**SD**	**N**	**Means**	**SD**	**N**
0.54759	0.14876	3	1.00000	0.79421	3	9.69506	2.51578	3

Table S2D statistical data of Figure1B

NC group			Control group			UHRF1-high group		
**Means**	**SD**	**N**	**Means**	**SD**	**N**	**Means**	**SD**	**N**
2.13812	1.09708	3	1.00000	0.10012	3	2.80093	1.74541	3

Table S2F statistical data of Figure3A

NC group			Control group			UHRF1-high group		
**Means**	**SD**	**N**	**Means**	**SD**	**N**	**Means**	**SD**	**N**
100%	0.11851	12	90.20%	0.058178	12	139.80%	0.11839	12
